# Policy and Linkages in the Application of a One Health System for Reporting and Controlling African Trypanosomiasis and Other Zoonotic Diseases in Zambia

**DOI:** 10.3390/pathogens11010030

**Published:** 2021-12-28

**Authors:** Gloria M. Mulenga, Boniface Namangala, Kalinga Chilongo, Lars Henning, Bruce Gummow

**Affiliations:** 1 College of Public Health Medical and Veterinary Sciences, James Cook University, Townsville, QLD 4814, Australia; lars.henning@jcu.edu.au (L.H.); bruce.gummow@jcu.edu.au (B.G.); 2 Department of Veterinary Services, Ministry of Fisheries and Livestock, Lusaka 10101, Zambia; kalinga.chilongo@mfl.gov.zm; 3 Department of Veterinary Services, Kakumbi Tsetse and Trypanosomiasis Research Station, P.O. Box 70, Mfuwe 10101, Zambia; 4 Department of Paraclinical Studies, School of Veterinary Medicine, The University of Zambia, Lusaka 10101, Zambia; b.namangala@unza.zm; 5 Faculty of Veterinary Science, University of Pretoria, Pretoria 0028, South Africa

**Keywords:** One Health, African trypanosomiasis, reporting structures, zoonotic diseases

## Abstract

The capacity to detect, control and manage emerging and re-emerging zoonotic diseases in Africa has been limited by a lack of utilisation of available reporting structures and policies to support programmes at national and local levels. This study explored the impact of the Zambian government policies on animal and human disease reporting and management and on One Health opportunities. An in-depth review and analysis of strengths, weaknesses, opportunities, and threats in the existing policies and reporting structures in the departments responsible for Veterinary Services, Health, and Wildlife, was conducted. According to our findings, sub-optimal implementation of existing policies related to the control of zoonotic diseases was impacting disease reporting, and reporting structures play an important role in effective and sustainable reporting of zoonotic diseases. Further, the study explored capacities and strategies in trypanosomiasis control as a case study that could prompt effective adoption of a One Health approach, and as such, the study suggests measures that could help to assess the performance of a One Health system in the control of African trypanosomiasis and other zoonotic diseases.

## 1. Introduction

The occurrence of human and animal African trypanosomiasis is associated with the presence of tsetse flies. Mammalian wild animals such as lions, buffalos, hippos, and elephants are the main reservoirs for the tsetse-transmitted trypanosome parasites [1,2]. However, an influx of people into tsetse infested areas has tended to increase the importance of humans and domestic animals as reservoirs of trypanosomes, and this is an increasing reason for concern [3,4,5]. The World Health Organisation (WHO) and Food and Agriculture Organisation (FAO) project that, in Africa, over 65 million people and 50 million head of cattle, respectively, are at risk of exposure to infection with African trypanosomiasis. However, due to poor and/or nonexistence of active surveillance for Human African trypanosomiasis (HAT) and Animal African Trypanosomiasis (AAT) in the affected countries, few cases of the disease are diagnosed and reported annually [6,7,8].

With about five-eighths (5/8) of Zambia estimated to be infested with tsetse flies, overall AAT prevalence in Zambia remains unknown. Based on trypanosomiasis studies conducted in different regions of the country, AAT prevalence in livestock ranges from 1% to as high as 90%. Over 60% of the country’s cattle population is under threat from trypanosomiasis with about 80% of the livestock owned by traditional farmers. The prevalence of trypanosomiasis in livestock and particularly in cattle has continued reporting alarming figures in affected areas [9,10,11]. According to data collected during a trypanosomiasis survey conducted in Mambwe district of eastern Zambia, AAT prevalence in cattle stood at 3.8% [12]. Meanwhile, the prevalence of HAT in Zambia as provided in the latest update for 2018 stood at 8.3%, higher than that of Malawi (5.7%) and the Democratic Republic of Congo (5.9%) [13]. Zambia, like many HAT endemic countries, faces several challenges in the successful implementation of HAT elimination programs. These include, among others, shortage of trained health workers in some areas, inadequate diagnostic and treatment centres, lack of more sensitive laboratory diagnostic techniques and shortage of trypanocides for effective treatment, which need to be instituted early enough to minimise serious drug reactions and mortality [13,14,15].

Most developing countries in Africa are faced with poor policy support with regard to animal disease surveillance [16]. Poor and/or inadequate policies and weak and unsustainable reporting structures limit capacity to detect and control emerging and re-emerging zoonotic diseases such as African trypanosomiasis in developing countries [6,17]. In sub-Saharan Africa, diagnostic capacity for many of the zoonotic diseases that are endemic in these countries is generally poor. According to studies conducted in Zambia, there is limited laboratory capacity for diagnosis of such diseases in literally all the provinces in the country [10,14,15]; thus, lack of effective diagnostic capacity and under diagnosis has been a critical weakness regarding effective treatment and control of infectious diseases. This challenge is mainly based on inadequate laboratory facilities and associated equipment and skilled laboratory personnel. This has affected the countries’ capacity and efforts to deal with re-emerging and emerging zoonotic diseases such as trypanosomiasis [10,14,15,18].

The global health security agenda specifically identifies One Health as an integral part of efforts to achieve health security against the threat of infectious diseases and other public health emergencies. Analysis by the World Bank suggests that given the high economic and health burden of zoonotic diseases, strengthening human and veterinary health capacity, to facilitate One Health approaches to disease prevention and control at the country level, could yield high returns on investment, averaging $30 billion per year [19]. Despite strong overall interest in the One Health approach, implementation at the country, local, and project level in Zambia remains limited.

This study compared and examined the policies and reporting structures in Departments of Veterinary Services, Health and Wildlife, in Zambia, in the context of the existing strengths, weaknesses, opportunities and threats, with the aim of gaining some insights into prospects for effective multi-sectoral and coordinated surveillance systems for zoonotic diseases. The study also examined existing opportunities and capacities among personnel and available operational provisions, in the Departments of Veterinary Services, Health and Wildlife, that could be used in a One Health approach to better manage and control trypanosomiasis and other zoonotic diseases.

## 2. Results

### 2.1. Reporting Structure in Relation to Occurrence of Animal Diseases

**Figure 1:**
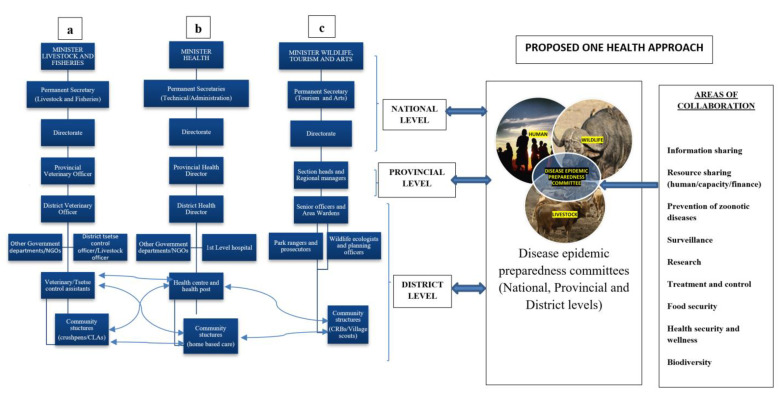
Zambian reporting structures and conceptual framework for the Ministries of Health, Livestock and Fisheries and Wildlife, Tourism and Arts showing areas where the One Health approach can be applied for the control of African trypanosomiasis and other zoonotic diseases. Horizontal bold double arrows indicate areas where the One Health approach at that level can be applied. Curved double arrows indicate areas where officers can brief each other on disease situation and response taken. (**a**): Reporting structure for Ministry of Fisheries and Livestock (**b**): Reporting structure for Ministry of Health (**c**): Reporting structure for Ministry of Wildlife, Tourism and Arts, respectively.

Control of animal diseases falls under the jurisdiction of the Department of Veterinary Services in the Ministry of Livestock and Fisheries (MLF). The ministry is headed by a Minister who undertakes the sourcing of funds and oversees the allocation of resources to the various programmes and activities in the ministry (Figure 1a). The controlling officer in the ministry is the Permanent Secretary (PS), under whom directors fall–and among these is the Director in the Department of Veterinary Services (DVS) that has two branches, each headed by a deputy director, i.e., ‘Veterinary Field Services’ and ‘Veterinary Research, Epidemiology and Information’. Each of the branches has two units, each headed by a ‘Chief Officer’. Under the ‘Veterinary Field Services’ branch, there is the ‘Veterinary Field Services Unit’ (VFSU) and ‘Tsetse and Trypanosomiasis Control Unit’ (TTCU). In the ‘Veterinary Research, Epidemiology and Information Branch’, there is the ‘Veterinary Research and Diagnostics Unit’ and the ‘Epidemio-surveillance and Information Unit’. In each of the units, there are ‘Principal Officers’ that report to the ‘Chief Officers’. With regard to the positions that fall below the ‘Principal officer’, it is only in the Veterinary Field Services Unit where there is a structure with officers stationed at the ministry’s offices in each province and district and also at the veterinary camp level in each district, i.e., Senior Veterinary/Tsetse Officers (at province level), District Veterinary/Tsetse Officers and Livestock officers (LOs) (district level) and, Veterinary Assistants (VAs) and Tsetse Control Assistants (TCAs) (at camp level). In the other units, ‘Senior Officers’ and ‘District Officers’ are strategically deployed only in selected provincial and district offices. In the case of TCAs and other personnel under the TTCU in the camps and districts, they are expected to also relay information (reports) on the occurrence of trypanosomiasis directly through the unit’s line of reporting.

Within the structure, it is the VAs and the TCAs that interact routinely with farmers and hence with livestock, and as such, it is these personnel that constitute the front-line workers and the first and most important sources of information on disease occurrence. They are also the first line of defence in the control and management of livestock diseases, i.e., they are expected to be the first to see indicators of disease (clinical signs), take the first possible/recommended interventions where feasible, and relay the necessary information (reports) urgently to their supervising officers (at district level) on suspected disease outbreaks and also routinely (e.g., monthly) on the general disease situation in their areas (camps) of jurisdiction (i.e., at district level). Some districts have trained Community Livestock Assistants (CLAs) who help report cases of animal disease that occur in their communities to the VAs (Personal communication, Mambwe District Fisheries and Livestock, 2019) [20,21]. Thus, the structure/system is such that information flow (reporting) on the occurrence of a disease such as trypanosomiasis is expected to start at the camp level (where there is routine interaction between farmers and the department’s camp personnel), flow upwards to the district officers and then to the provincial officers (for scrutiny/evaluation and quality control), and later, transmit to the Chief Officers in the Veterinary Field Services and Epidemio-surveillance and Information units of the directorate of Veterinary Services.

However, regarding animal trypanosomiasis, active surveillance is carried out intermittently by the TTCU, and this information is made available through the specific reports.

### 2.2. Reporting Structure in Relation to Occurrence of Human Diseases

The Ministry of Health (MOH) is responsible for reporting all human diseases occurring in Zambia. Just like the MLF, the MOH is also headed by a Minister (Figure 1b). Under the minister, there are two Permanent Secretaries (administration and technical services). Each Permanent Secretary also has several directors and chief staff herein referred to as the directorates. The directorate gathers all reports from the provinces. The ten directorates at MOH are clinical care, public health, finance, human resources, policy and planning, infectious disease, monitoring and evaluation, nursing, and quality improvement and performance. The provinces are headed by the Provincial Health Directors (PHDs), who also have principal and senior officers under them. Under the PHDs are District Health Directors (DHDs) who receive all reports from the hospitals, health centres, health posts, other government departments and other Non-Government Organisations (NGOs) operating in their districts. Some districts also have trained community health workers and, in some places, community health assistants who work hand in hand with their local health centres or health posts [21,22]. Similarly, with the MLF, the structure also allows the flow of information from communities through interactions with community structures.

### 2.3. Reporting Structure in Relation to Occurrence of Wildlife Diseases

The Department of Wildlife and National Parks, formally known as Zambia Wildlife Authority (ZAWA), falls under the Ministry of Tourism and Arts (Figure 1c). The department is supervised through the Head office, Regional offices, and the Area Management Units. The Head Office basically provides supervisory roles and backstopping services to the Regional Offices and the Area Management Units. The Regional Offices also supervise the Area Management Units under their jurisdiction and implement some activities. The Area Management Units mainly implement the department’s activities throughout the country. At the Head Office, which falls under the Permanent Secretary, there is a management structure, which is headed by a Director General who has the overall responsibility for the day-to-day management of the department. There is a line management of six directorates, namely, Conservation and Management, Research, Planning and Information, Commercial Services, Game Management Areas, Finance and Corporate Services, and Legal Counsel. These together with the Administration and Human Resources Manager, Head Intelligence and Investigations, Chief Internal Auditor and Projects Coordinator comprise the senior management structure of the department. The Department of Wildlife is run under a decentralised system, with four (4) geographical regions (Eastern, Western, Northern and Southern and with their offices in Mfuwe, Mumbwa, Kasama and Mazabuka, respectively). Each region is headed by a Regional Manager who is assisted by an Area Warden, a Regional Accountant, an Extension Officer, Park Ranger and Senior Investigations Officer. Although the organisational structure is said to be decentralised most of the management decisions (i.e., procurement, disbursement of funds, etc.) are still very much centralised at headquarters where most decisions are made [23,24]. The department also has community structures, which allows the flow of information from the communities to the regional offices and management.

### 2.4. SWOT Analysis—Reporting Structure on Occurrence of Animal and Human Diseases

The results of the SWOT analysis are as shown in Table A1 (Appendix A). In the reporting structures, there were clear similarities between the Department of Veterinary Services and Health, compared to the Department of National Parks and Wildlife, while some elements were common in all three government institutions under review.

### 2.5. Questionnaire Survey

**Figure 2:**
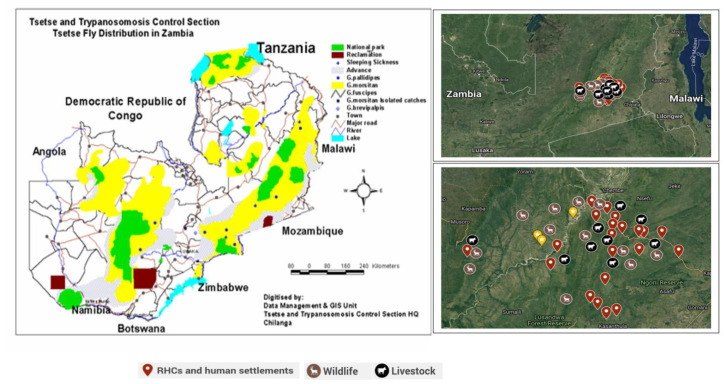
Tsetse and trypanosomiasis distribution in Zambia. Source: (Tsetse Control Section, Zambia, 2018). Inserts showing locations of Rural Health Centres (RHCs) visited and distribution of wildlife and livestock in the study area. Source: (Mulenga, 2021, Google Maps).

In total, 21 health centres and health posts from Mambwe district were involved in our survey, namely, Kamoto, Chilanga, Mphata, Kakumbi, Airport, Masumba, Nyamaluma, Kasamanda, Ncheka, St. Lukes, Malama, Kamubaba, Chikowa, Nyakatokoli, Lupande, Lusamdwa South, Chipako, Jumbe, Chisengu, Jumbe and Mphomwa (Figure 2). Only healthcare personnel that were currently working and were present at the centres were interviewed. Due to low staffing levels from the Department of Veterinary Services, all key personnel present in the district were interviewed, while interviews from the Department of National Parks and Wildlife were focused on officers from Chinzombo offices in Mfuwe. The numbers of key personnel for disease control available at the centres visited (Table 1) were as reported by personnel operating the centres. It is important to note that only officers that agreed to be interviewed were involved in the study.

During interviews, responses on the availability of parasitological and molecular tools (Table 2) that could be useful for both passive and active surveillance of trypanosomiasis in man and animals were recorded and are indicated below.

Respondents from the Veterinary Department indicated they received financial support for the control of trypanosomiasis, while respondents from the Departments of Health and Wildlife reported no financial support for trypanosomiasis control (Table 3). In the same manner, the Veterinary Department reported undertaking more surveys and surveillance for tsetse and trypanosomiasis (T & T) as compared to their health and wildlife counterparts (Chi-square, *p* = 0.001) (Table 3). On the other hand, the Department of Wildlife indicated that they collaborated more with other departments and NGOs than the Health and Veterinary Departments (Chi-square, *p* = 0.04) (Table 3).

Type of collaboration required as indicated by the Departments of Veterinary, Health and Wildlife during the survey included the following: staff training and capacity building, disease awareness and management and disease diagnosis.

## 3. Discussion

The results of the review of reporting structures for the Zambian Departments of Veterinary Services, Health, and National Parks and Wildlife (Table A1) indicate opportunities that may exist for improved disease reporting and management. The study identified existing links in reporting systems (Figure 1) that could be used to provide a more holistic response to emerging and re-emerging livestock, human and wildlife diseases [20,21,23,25]. The Zambian Departments of Health and Veterinary Services have been using the Public Health Act and Animal Health Act of the Laws of Zambia as major policies to guide the provision of human and animal health care services, respectively [20,21]. These acts have clear statements on the reporting procedures of notifiable diseases. The study also found similarities between the organisational structures of the Departments of Veterinary and Health (Figure 1) that could be utilised in disease reporting and the adoption of a One Health system. On the other hand, the Zambian Wildlife Act has no mention of reporting notifiable diseases despite most zoonotic diseases having a wildlife origin [7]. The Wildlife Act has instead focused on management and protecting wildlife areas whilst overlooking wildlife disease management [23].

Findings from the survey indicate limited government financial support for the three government departments to undertake surveys/surveillance for the control of trypanosomiasis and the need to strengthen collaboration between sectors for disease control and management. A previous study [14] conducted in the area re-affirms the absence of financial support to manage trypanosomiasis whilst similar diseases such as malaria, HIV/AIDS and tuberculosis remain on the government’s funding priority list. Such limited priorities in areas of livestock/wildlife disease support from local authorities has a negative impact on zoonotic disease response as infection rates in either domestic or wild animals can be early predictors of transmission risks to humans [26,27,28]. According to findings by Mandyata et al. [22], several challenges, including human resources, poor infrastructure and coordination, hamper effective response to re-surging diseases. Our study confirms these gaps in human/financial resources and laboratory tools that could be used in an ideal setting for trypanosomiasis management and other zoonotic diseases. Sharing human capacities and collaboration on trypanosomiasis awareness and management as suggested by respondents from our survey could help achieve health security against the threat of other infectious diseases [22,25].

Governments of endemic trypanosomiasis areas are, however, overwhelmed with the costs attached to the sustainable control of trypanosomiasis, thus, making its control difficult [29]. The high cases of HAT in Zambia compared to neighbouring Malawi and the Democratic Republic of Congo could be mainly related to spillovers from wildlife and livestock reservoirs that dwell within human settlement areas, as observed from our survey map in Figure 2. Reinforced passive surveillance, scaling up of active surveillance and sustained control efforts, backed up by an adequate surveillance system in Malawi and the Democratic Republic of Congo has resulted in the reduction in HAT cases [13]. Such lessons learnt from Zambia’s neighbouring countries can be adopted to improve the HAT and AAT situation locally.

The Zambian government has made efforts to control tsetse and trypanosomiasis, but due to financial limitations and other disease burdens, trypanosomiasis control programmes have not been sustained [30,31]. This has resulted in tsetse re-invasions and disease flareups even in areas where control was once undertaken. From the time of British colonial rule through independence to date, Zambia has used several approaches to try and combat trypanosomiasis. These include ground spraying, occasional use of sequential aerosol technique (SAT), the use of curative and prophylactic trypanocides, the use of odour baited targets, traps, and live baits [13,31,32].

Currently, in consideration of past lessons learnt and in adopting the approach of the African Union’s Pan African Tsetse and Trypanosomiasis Eradication Campaign (AU-PATTEC), Zambia has adopted the principle of an area-wide integrated pest management, which is based on interventions against trypanosomiasis [31]. However, the control of the tsetse vector in protected areas and game reserves could be more complicated due to conservationist, ecological, and environmental considerations [33]. Current methods for tsetse control include non-insecticidal (bush clearing, the sterile insect technique, and the use of insect symbionts), and insecticidal methods (odour baits, SAT and ground spraying). Tsetse infested areas are categorised as low, medium, and high priority areas to determine the type of intervention to be employed [13,27,34].

Through review of reporting structures for the Departments of Veterinary Services, Health, and Wildlife, as shown in Table A1 (Appendix A), we identified areas through which departments could maximise resources, share information, and collaborate. These areas exist at national, provincial and district levels, as indicated by bold horizontal lines in Figure 1. The literature also revealed the existence of epidemic preparedness committees at national, provincial and district levels [22] (Ministry of Livestock and Fisheries reports). The committees comprise members from government departments, which include, among others, Health, Veterinary, Wildlife, Agriculture Lands and Natural resources, Education, Community Development and Partnering Non-Government Departments. These committees, if utilised effectively, provide a good platform for reporting zoonotic diseases and their status. We advocate that committees can also be used to promote and drive One Health strategies that will promote biodiversity, food security, safe environment, information and resource sharing, human and animal health, as well as strengthen the collaboration and coordination between sectors in order to improve the prevention and control of zoonotic diseases [25,35] (Figure 1). The chairpersons for these committees, who are provincial and district administrative officers, respectively, can spearhead and direct solutions for the implementation of a One Health approach in their respective areas.

An analysis of reporting structures for the Departments of Veterinary, Wildlife, and Health reveals that each department had its strengths, weaknesses, opportunities, and threats, as indicated in Table A1 (Appendix A). However, some issues were common across the departments studied. All three reporting structures allowed for interactions between senior and junior officers, but the culture of not bypassing immediate supervising officers created a challenge in the timely reporting of disease incidences. The limited and low levels of key personnel for disease diagnosing and surveillance, as indicated from the survey data, created a gap in the reporting system. The non-availability of senior personnel especially at the district level limits reporting capacities of junior officers who may not be experienced enough. Our results indicate that against a population of over 96,000 (Zambia, Central statistics projections 2019), the Mambwe district had 20 health posts and a hospital with a bed capacity of 170 (personal communication). The district is managed by only four doctors who are overwhelmed with work, thus impacting negatively on their capacities to service delivery [14]. In the same manner, the Veterinary Department was the worst hit in terms of staffing levels. The department had no veterinary officer and only six veterinary assistants who are field officers to cover and manage livestock diseases in a district with an area size of 4480 km squared and over 18,000 households (Zambia, Central statistics data, Mambwe district, 2015). The absence of key personnel responsible for disease surveillance and reporting at the grass-root level could threaten the effective reporting of trypanosomiasis and other animal and human diseases.

The reporting structure for the Wildlife Department (Figure 1) is long, which may impact timely reporting, especially if some positions are vacant. The tough training and military culture incorporated in the management of National Parks and Wildlife may also contribute to the rigidness of the structure, thus affecting the processing time of reports and information sharing. There is a need to shorten the reporting structure, which will promote interaction and information sharing, thus increasing efficiency of disease reporting.

In general, the study identified levels, as indicated in Figure 1, within reporting structures under review, that can be platforms for supporting more collaboration, information and capacity sharing between departments. Interactions at national, provincial and district levels may also allow for the development of policies that will promote a collective approach in the management and control of zoonotic diseases [16,25,36]. To overcome the resource challenge as indicated from our findings, resources could be saved and re-allocated to other activities through combining human and livestock/wildlife activities, e.g., concurrent sampling of both human and animal subjects, sharing of diagnostic capacities and cross-training of the staff of the Departments of Veterinary, Wildlife, and Human Health. Introducing more holistic approaches and policies for cross reporting within systems may be a more sustainable approach towards achieving a One Health approach. The recent creation of the Zambia National Public Health Institute (ZNPHI), under the Zambian MOH [37] has been a step further into improving reporting systems of diseases that are of public health interest. The Tropical Diseases Research Centre (TDRC), an initiative of the WHO, also under the Zambian MOH and the Central Veterinary Research Institute (CVRI) under the Zambian MLF are other institutions that can be strengthened for effective management of zoonotic diseases by incorporating a multi-sectoral coordination approach. Broadening the capacities of these existing Institutions will be a better approach towards effective management and control of zoonotic diseases.

## 4. Materials and Methods

The provisions in the Zambian Public Health Act, Animal health Act and Wildlife Act were evaluated in the context of providing the key elements on national policies and on animal disease reporting systems in Zambia. Organisational structures of each of the departments responsible for Veterinary Services, Health, and Wildlife, were also examined in relation to disease reporting systems (Figure 1). An online search was conducted in December 2020 using One Search hosted by James Cook University, Townsville, Australia, using the following keywords: “National Policy” AND, OR “Animal Disease Reporting” AND, OR “Human Disease Reporting” AND, OR “Zambia”. A systematic review of reporting structures for the Departments of Veterinary Services, Health, and Wildlife in Zambia, was conducted. Analysis of the strengths, weaknesses, opportunities, and threats (SWOT analysis) in existing reporting structures and policies with respect to trypanosomiasis was applied for the departments under review.

In Zambia, personnel that undertake disease diagnosis are of various training/professional backgrounds of human and animal health service providers. An interview-based questionnaire study targeting all officers based in Mambwe in Zambia was conducted within the Veterinary Department (9), Wildlife Department (15), and Health Department (21). The interviews looked at the management and control of African trypanosomiasis in their respective departments. To conduct the study, written informed consents were obtained from all respondents before administering the questionnaires. Participation in the study was voluntary, and participants were free to withdraw from the study without giving any reasons. Information sheets (Appendix B) were provided for each recruited participant explaining the aims, benefits of the study, and possible risks. The focus of the questionnaire (Appendix C) was on the departments’ ability to detect African trypanosomiasis and any of the other zoonotic diseases known to be prevalent in the area. Included were questions on funding provisions for trypanosomiasis control and management in their departments, and on any existing collaboration with other government departments or with other organisations/institutions in general and the reporting structure/system in the respective departments in relation to occurrence of trypanosomiasis in the area.

### Data Analysis

We conducted a SWOT analysis [36] of policies and reporting structures in each of the departments. The data from the interviews were stored in an MS Excel file and later exported to IBM SPSS Statistics 27, where it was summarised as frequencies and percentages and analysed using descriptive statistics. The Chi square test was used to compare proportions between departments. For each analysis, *p* values < 0.05 were considered statistically significant. Fisher’s exact test was used to compare proportions between districts where expected values or responses were less than five.

Ethical clearances were obtained from James Cook University (H7226 and A2498) and the Zambian Ethics Committee (Ref. No. 2018-Oct001), and the research was approved by the Zambia National Health Research Authority

## 5. Conclusions

Coordinated surveillance systems within available organisational structures could play a key role in disease reporting and have the potential to impact the reporting of emerging and re-emerging diseases. A better One Health system could be applied in Zambia and other countries in the region and beyond by strengthening links for collaboration and coordination at national, provincial and district levels between sectors (health, veterinary, wildlife and natural resources) and by creating improved reporting links within available reporting structures that will promote interactions and provide a more holistic response to disease control. This can be achieved through already existing institutions such as CVRI, ZNPHI, and TDRC, as well as through epidemic preparedness committees. With a slight shift in focus to include zoonotic diseases, the ZNPHI could provide the platform for disease reporting between partnering departments.

More efficient use of existing capacity by implementing a One Health approach is possible between sectors. For example, in areas where veterinary services have laboratory capacity, samples from the Departments of Health and Wildlife can be sent to veterinary facilities for analysis and vice versa. In addition, community awareness programmes for zoonotic diseases and collaborated staff training/upscaling of skills related to veterinary, health and wildlife can be collaborative to save resources. The digitalisation of records for information sharing through national, provincial and district epidemic preparedness committees could be carried out in partnership to create a more efficient response system. To support sustainable zoonotic disease control approaches that can be implemented at national, provincial and district levels, new policies will need to be developed in the future.

## Figures and Tables

**Table 1:** Demographics of key personnel involved in managing trypanosomiasis and other zoonotic diseases in the Mambwe district of Eastern Zambia in February 2020.

	Profession	Qualification	Positions Present at Time of Study
Health	Medical officers	Degree	4
	Clinical officers	Diploma	19
	Nurses	Diploma	95
	Environmental health technicians	Certificate	20
	Laboratory technicians	Diploma	13
Veterinary Services	Veterinary officers	Degree	0
	Biologists	Degree	1
	Livestock officers	Diploma	1
	Livestock technicians	Diploma	1
	Veterinary assistants	Certificate	6
	Laboratory technicians	Diploma	0
Wildlife and National Parks	Veterinarians	Degree	1
	Ecologists	Degree	1
	Laboratory technicians	N/A	0

**Table 2:** Results on availability of laboratory tools that could be used to diagnose trypanosomiasis and other zoonotic diseases.

Diagnostic Tool	Health	Veterinary	Wildlife and National Parks
Microscopy	Present	Present	Present
Rapid test kits	Absent	Absent	Absent
PCR	Absent	Present	Absent
LAMP	Absent	Absent	Absent

Abbreviations: PCR: Polymerase Chain Reaction; LAMP: Loop-Mediated Iso-Thermal Amplification.

**Table 3:** Results of responses on tsetse and trypanosomiasis control and management and options for collaboration.

	Health (*n* = 21)			Veterinary (*n* = 9)			Wildlife and National Parks (*n* = 15)		
	Yes	No	Do Not Know	Yes	No	Do Not Know	Yes	No	Do Not Know
1. Does the centre receive financial support for trypanosomiasis?	0	19 (90.5%)	2 (9.5%)	8 (88.9%)	1 (11.1%)	0	0	12 (80%)	3 (20%)
2. Does your department undertake trypanosomiasis surveys/surveillance?	6 (28.6%)	12 (57.1%)	3 (14.3%)	9 (100%)	0	0	2 (13.3%)	11 (73.3)	2 (13.3)
3. Does your department work with other GRZ/NGOs on trypanosomiasis issues?	4 (19%)	17 (81%)	0	4 (44.4%)	4 (44.4%)	1 (11.1%)	12 (80%)	2 (13.3)	1 (6.7%)

Abbreviations: GRZ: Government of the Republic of Zambia; NGO: Non-Government Organisations.

## Data Availability

Not applicable.

## Appendix A

**Table A1:** Summary of SWOT analysis for reporting structures for the Departments of Veterinary Services, Health and National Parks and Wildlife.

Internal		External	
Strengths	Weaknesses	Opportunities	Threats
Veterinary Services (Figure 1a) The structure was flexible and allowed for interactions among officers—for example, VAs and TCAs could report directly to DVOs. In the same manner, LOs could report directly to the senior veterinary officers in the province. The TTCU is mandated to specifically oversee all tsetse and trypanosomiasis control and management programmes in the country. All heads of units in the Department of Veterinary Services interact frequently, and this facilitates sharing of information on the occurrence of livestock diseases. Directors from the livestock sector at the national level also interact frequently and share information. Personnel at district and provincial levels interact routinely with their colleagues from other ministries and NGOs, and this allows for information sharing	Extension officers cover very large areas, and this made it difficult to achieve timely reporting of disease occurrence. The flexibility of reporting to senior veterinary officers directly creates gaps in knowledge among immediate staff. The process of replacing deceased/retired officers is slow, resulting in vacant positions. The structure does not allow for position funding to ensure timely filling of vacancies. Shortage of staff makes work overwhelming for available officers.	Some veterinary camps could be divided into smaller units per VA or TCA to facilitate more effective coverage by the Vas and TCAs efficiently. **A network of reporting and information sharing needs to be enhanced between staff (including staff from Health and Wildlife Departments) and the community by creating better extension methodologies**. **Additional positions could be created in the structure to improve on the effectiveness of reporting on trypanosomiasis and other zoonotic diseases occurrences**. Levels of funding to the TTCU could be improved, increasing capacity for the unit to undertake collection of relevant data/information more routinely and more effectively on tsetse and trypanosomiasis in the country. **The mandate/role of the TTCU could be increased/enhanced to include more elements in the One Health approach to addressing the trypanosomiasis problem and other zoonotic diseases**. Improvement of salaries and other working conditions for personnel in the department could encourage/enhance better performance of personnel in the department.	Employment of extension personnel at VA/TCA and related levels occurs, and this diminishes prospects for effective/beneficial interactions with farmers regarding collection of information on disease occurrence. Some districts such as Mambwe do not have DVOs, which makes gaps in reporting and decision making. Slow recruitment of staff and non-availability of funds has led to prolonged vacancies in key positions. Shortage of staff makes work overwhelming for available officers. Prolonged poor funding for the ministry. Lack of operational funds for the T & T has created a group of unmotivated staff who must perform other duties of the department. Crushing economy and stagnant salaries. Reduced political will.
Health (Figure 1b) The structure is flexible and allows interactions between junior and senior officers. Department and section heads interact frequently, enabling disease information sharing Permanent secretaries and directors at the national level interact frequently and share information. The ministry is the secretariat for epidemic preparedness meetings at national, provincial and district levels. Staff at both district and provincial levels interact with their colleagues from other ministries and NGOs through the epidemic preparedness, which allows for networking and information sharing. Strong interaction between health workers and communities through health centre committees at local levels.	Some health centres/posts have a large coverage therefore reduce effective service delivery. The flexibility of reporting to senior officers directly creates gaps in knowledge among immediate staff. Some districts such as Mambwe do not have a district hospital, which has overwhelmed the mission hospital that has limited bed and staff capacity. Epidemic preparedness committees prioritise and are driven to report on diseases of political interest and affect the majority, while diseases that affect the voiceless poor, remote communities are not given much attention. Shortage of staff makes work overwhelming for available officers. Focus areas are determined at the national level, which may not be priorities in all districts.	**The building of more health posts across the country will enhance service delivery and increase points of contact and interaction. This will also create opportunities for active epidemiological assessments of zoonotic diseases**. **Creation of district hospitals and converting some hospitals at the province to level 2 or 3 hospitals could provide relief for health centres/posts. District hospitals could provide common public health services conveniently**. **Data collection takes place at all levels, which can enhance information sharing between line ministries and departments with emphasis on zoonotic diseases**. **Opening of more health training facilities, both public and private, will provide person power to fill key positions/vacancies**. Improved salaries and attractive conditions of service for medical personnel will motivate and encourage them to join the public sector.	Some health workers are new and inexperienced. Majority of experienced health workers have fled the country in search of greener pastures. Slow recruitment of staff and non-availability of funds has led to prolonged staff vacancies in key positions at health facilities. Crushing economy and stagnant salaries. Reduced political will. Erratic and inadequate funding to health resulting in poor performance of the sector. Inadequate laboratory equipment and reagents to detect disease outbreaks early.
National Parks and Wildlife (Figure 1c) The department operates under a decentralised structure. The department works hand in hand with other wildlife conservation organisations enhancing the control, management, conservation and administration of national parks, bird sanctuaries, wildlife sanctuaries and Game Management Areas (GMAs). Through partnership with local communities (CRBs), responsibilities of management in game management areas are shared. Community networks allows sensitisation and education of the public on the necessity of wildlife conservation, and the importance of wildlife to foster appreciation of the economic value of wildlife. Networking and information sharing is enhanced through staff interaction at all levels of management	Though the structure is decentralised, decisions and allocation of financial resources is still conducted at headquarters. The department, which was formally an authority and managed by a board, is now a government department and operations are now dependant on irregular government funding. Poaching has negatively impacted on the department’s operations and is a threat to tourism. Corruption when dealing with recruitments, prosecutions, and issuance of licenses. The process of replacing deceased/retired officers is slow, resulting in vacant positions. The structure is large.	Decision making and financial allocation of funds should be decentralised. Improved funding will strengthen operations and enhance control, management, and conservation of wildlife. **Improved funding will also strengthen active surveillance of zoonotic diseases originating from wildlife**. **More smart partnerships need to be created with other government departments, NGOs and the private sector**. **Community awareness and sensitisations need to be enhanced and be undertaken in collaboration with other government departments and NGOs**. Community empowerment programmes to be created to reduce poaching. Improved salaries for wildlife officers. **Create policy to retain key positions**. **Create sections within the department that could be responsible for addressing zoonotic diseases, including trypanosomiasis, thereby promoting the One Health approach**.	Recruitments are slow and politically driven. Limited funding has created a lot of vacancies and weakened operations. Reduced political will Crushing economy and stagnant salaries. The large structure is a challenge to timely reporting

Abbreviations: VAs: Veterinary Assistants; DVOs: District Veterinary Officers; LOs: Livestock Officers; TCAs: Tsetse Control Assistants; TTCU: Tsetse and Trypanosomiasis Control Unit; T & T: Tsetse and Trypanosomiasis; CRBs: Community Resources Boards). Bold and highlighted ‘opportunities’ indicate areas where a One Health approach can be applied.

## Appendix B

INFORMATION SHEET

PROJECT TITLE: The control of Bovine and Human African Trypanosomiasis and role tsetse endosymbionts play in disease transmission in endemic areas of Zambia.

You are invited to take part in a research project that aims at evaluating and identifying strategies and measures that are economically important in the control of African trypanosomiasis. The study will help in improving detection of Nagana (AAT) in cattle and sleeping sickness (HAT) in people in endemic areas of Zambia. This will help authorities to more rapidly treat infected people and cattle. The study will also help in our knowledge of the role and importance different control strategies play in the control of Animal Trypanosomiasis so that we can identify what will be the most cost effective way of controlling this disease in your region of Zambia. This could help make the control of the disease more affordable in your region. The study is being conducted by **Gloria Mulenga** and will contribute to the attainment of a **Doctor of Philosophy Degree (PhD)** in **Epidemiology** at James Cook University in Australia.	
If you agree to be involved in the study, you will be invited to be interviewed through a questionnaire. The interview, with your consent, should only take approximately 45 min of your time. The interview will be conducted at your home, or a venue of your choice. The questionnaire that you will be requested to complete, asks you about your personal details including the level of your education. It also asks you questions on farm structure and income, trading practices and interaction.	
Taking part in this study is completely voluntary and you can stop taking part in the study at any time without explanation or prejudice.	
Your responses will be non-identifiable so no one will know who gave the information to us. The data from the study will be used in research publications and reports to be published by the principal investigator and other collaborators through **James Cook University**, **University of Pretoria**, **Zambian Government and University of Zambia**. You will not be identified in any way in these publications.	
If you have any questions about the study, please contact—**Gloria Mulenga (Principle Investigator) and Bruce Gummow (Supervisor)**.	
**Principle Investigator:** **Gloria Mulenga** **Department of Veterinary Services** **Ministry of Fisheries and Livestock** **Republic of ZAMBIA** **Phone: +61(07)4781 6903** **Mobile: +260 977628915** **Email:** gloria.mulenga@my.jcu.edu.au	**Australian Investigator:** **Name: Bruce Gummow** **College of Public Health**, **Medical and Veterinary Science** **James Cook University**, **AUSTRALIA** **Phone: +61(07)47814071** **Mobile:** **Email:**bruce.gummow@jcu.edu.au

If you have any concerns regarding the ethical conduct of the study, please contact:

Human Ethics, Research Office

James Cook University, Townsville, Qld, 4811

Phone: (07) 4781 5011 (**ethics@jcu.edu.au**)

(Chairperson), ERES CONVERGE IRB; 33 Joseph Mwilwa Road Rhodes Park; LUSAKA; Tel: 0955 155633/4; Mobile: 0966 765503; E. Mail: **eresconverge@yahoo.co.uk**

## Appendix C

QUESTIONNAIRE SURVEY

THE CONTROL OF AFRICAN TRYPANOSOMIASIS AND THE ROLE TSETSE ENDOSYMBIONTS PLAY IN DISEASE TRANSMISSION IN ENDEMIC AREAS OF EASTERN ZAMBIA

QUESTIONAIRE ID: …………………………………… NAME OF RHC: ………………………………………… GPS COORDINATES: ……………………………………					
INSTRUCTIONS:					
1.No name should appear on/and or in this questionnaire. 2.Answer all the questions. 3.Tick √ in the space provided next to your choice. 4.Write in provided space wherever appropriate. 5.Use a pen/pencil in the questionnaire.					
SECTION A: DEMOGRAPHIC DATA			FOR OFFICIAL USE		
1 Occupation/position of the respondent at the center (In-charge)					
(1) Medical Officer (2) Clinical Officer (3) Nurse (4) Laboratory Technician (5) Environmental health technician (6) Other (specify)					
2 Highest level of education of respondent					
(1) Degree (2) Diploma (3) Certificate (4) Other (specify)					
3 Indicate numbers of staff at the clinic with the following occupations (Write in the space provided)					
(1) Doctors (2) Clinical Officer (3) Nurses (4) Laboratory Technician (5) Environmental health technicians (6) Other (Specify)					
4 For how long have you being working in this district?					
(1) Less than 5 years (2) Between 5 and 10 years (3) More than 10 years					
SECTION B: CAPACITY TO MANAGE SLEEPING SICKNESS/AAT					
5 Is tsetse transmitted sleeping sickness/AAT a problem within your community?					
(1) Yes		(2) No		(88) Don’t know	
6 What do you think plays a role in sleeping sickness/AAT transmission?					
(1) Wildlife	(2) domestic animals		(3) Tsetse		(88) Don’t know
7 From your knowledge, what signs and symptoms does a suspected sleeping sickness patient/AAT animal present themselves with?					
(1) Sleeping disorder (2) General body pains/extreme fatigue/severe headache (3) Chancre-swelling at site of tsetse bite (4) Puffy swollen face (5) Severe fever/history of taking anti-malarial drugs with no relief (6) Skin rash (7) Loss of weight/appetite (8) Anemia (9) Others					
8 Have you ever encountered a case of sleeping sickness/AAT at this health center?					
(1) Yes			(2) No		
If NO skip to Question 9					
9 From hospital/station records, how many cases of sleeping sickness/AAT has your Centre reported? (Ask to see records if available)					
(1) Last 12 months		(2) Last 5 years		(3) Last 10 years	
10 Does your department undertake surveys/surveillances for sleeping sickness/AAT within your catchment areas?					
(1) Yes		(2) No		(88) Don’t know	
If yes, how often?					
(1) Quarterly		(2) Annually		(3) Whenever resources available	
11 Has any officer at this Centre received any special training on sleeping sickness/AAT diagnosis/management?					
(1) Yes		(2) No		(88) Don’t know	
12 Does the Centre have a laboratory equipped with the following facilities?					
(Tick √ in the box next to your choice)					
(1) Standard diagnosis equipment/materials(Microscope, centrifuge, Giemsa stain, slides, capillary tubes)					
(2) Rapid test kits					
(3) Molecular technique facilities (PCR, LAMP)					
(4) None of the above					
13 Does the center receive any financial support specifically for the management of sleeping sickness/AAT?					
(1) Yes		(2) No		(88) Don’t know	
If yes, tick below					
(1) GRZ		(2) Private Sector		(3) Other	
SECTION C: KNOWLEDGE AND COLLABORATION					
14 Are you aware of the occurrence of AAT in your area?					
(1) Yes		(2) No		(88) Don’t know	
15 Do you think AAT is a problem in your area?					
(1) Yes		(2) No		(88) Don’t know	
16 Does your center work with officers from the Veterinary/Health/Wildlife department on issues related to sleeping sickness and AAT?					
(1) Yes		(2) No		(88) Don’t know	
If yes, which area of collaboration below?					
(Tick √ in the box next to your choice)					
(1) Community awareness					
(2) Staff training and capacity building					
(3) Laboratory diagnosis					
(4) Other (Specify)					
17 Does your department in your catchment area work with officers from the other government/non-governmental departments on issues related to sleeping sickness and AAT?					
(1) Yes		(2) No		(88) Don’t know	
If yes, which area of collaboration below?					
(Tick √ in the box next to your choice and indicate name of organization)					
(1) Community awareness					
(2) Staff training and capacity building					
(3) Laboratory diagnosis					
(4) Other(Specify)					
If no, would you want collaboration?					
(1) Yes			(2) No		

THE END

THANK YOU FOR YOUR COOPERATION

NOTE the following differences in the questionnaire for the Department of Veterinary Services:

SECTION A: DEMOGRAPHIC DATA		FOR OFFICIAL USE	
1 Occupation/position of the respondent			
(1) Veterinary Officer	(2) Tsetse control Officer	(3) Laboratory Technician	(4) Other (specify)
2 Indicate numbers of staff at the facility with the following occupations			
(Write in the space provided) SKIP Question if you are the only staff			
(1) Doctors (2) Biologists/Scientist (3) Field assistants (4) Laboratory Technician (5) Other (Specify)			

NOTE the following differences for the questionnaire for the Department of National Parks and Wildlife:

SECTION A: DEMOGRAPHIC DATA		FOR OFFICIAL USE	
1. Occupation/position of the respondent			
(1) Veterinary Officer	(2) Ecologist	(3) Laboratory Technician	(4) Other (specify)
2 Indicate numbers of staff at the facility with the following occupations			
(Write in the space provided) SKIP Question if you are the only staff			
(1) Doctors (2) Ecologist/Scientist (3) Field assistants (4) Laboratory Technician (5) Other (Specify)			
